# 55 year-old Female with Hematuria

**DOI:** 10.5811/cpcem.2020.2.46271

**Published:** 2020-04-23

**Authors:** Drew A. Long, Brit Long

**Affiliations:** San Antonio Military Medical Center, Department of Emergency Medicine, Fort Sam Houston, Texas

**Keywords:** hematuria, flank pain, renal cell carcinoma

## Abstract

**Case Presentation:**

A 55 year-old female presented to the emergency department with left sided abdominal pain and hematuria. Computed tomography scan of her abdomen and pelvis demonstrated a large left renal mass with extension into the left ureter, left renal vein, and inferior vena cava. She was admitted and treated for presumed renal cell carcinoma (RCC).

**Discussion:**

RCC may present with abdominal or flank pain and hematuria, but more commonly presents with vague symptoms. RCC should be suspected in a patient presenting with hematuria and abdominal or flank pain, especially if vague symptoms such as fatigue or anorexia are also present.

## CASE PRESENTATION

A 55 year-old female with a history of lymphoma, paroxysmal nocturnal hematuria, and undifferentiated renal masses presented to the Emergency Department with left sided abdominal and flank pain, fatigue, and hematuria. Abdominal examination demonstrated mild left upper quadrant tenderness. Complete blood count revealed a hemoglobin of 6.6 grams per deciliter and platelet count of 5,000 per microliter. Urinalysis demonstrated large (3+) blood and >182 red blood cells per high-powered field. Computed Tomography (CT) scan with intravenous (IV) contrast of her abdomen and pelvis was obtained (Image).

## DISCUSSION

CT scan was notable for an 8.2-centimeter necrotic mass in the left kidney with extension into the left renal vein, inferior vena cava (IVC), and left ureter. The patient was admitted to the hospital and transfused with 2 units of packed red blood cells and 1 unit of platelets. Oncology and urology services were concerned for renal cell carcinoma (RCC). The patient was scheduled for outpatient palliative radiation therapy and started on rituximab and eculizumab.

RCC is the most common type of kidney cancer in adults, responsible for 90–95% of cases.^1^ The classic triad of hematuria, flank pain, and a palpable flank mass occurs in 5–10% of cases.^2^ When present, this triad indicates a more advanced stage of the disease. More commonly, patients with RCC present with nonspecific symptoms such as fatigue, anorexia, weight loss, or fever of unknown origin.^3^

**CPC-EM Capsule**

What do we already know about this clinical entity?

Renal cell carcinoma (RCC) is the most common type of kidney cancer in adults. It may present with hematuria or flank pain, but more commonly presents with vague symptoms.

What is the major impact of the image(s)?

This image depicts RCC invading surrounding anatomic structures, leading to the clinical manifestation of hematuria.

How might this improve emergency medicine practice?

Emergency clinicians must suspect this diagnosis in adult patients with vague symptoms, especially if they have hematuria or flank pain.

It is estimated that RCC invades the IVC and forms a venous tumor thrombosis in up to 10% of cases,^4^ as seen in the presented patient. CT scan with IV contrast is highly sensitive for detecting both RCC and invasion into either the ureter or IVC.^5^ The Emergency Physician must consider this diagnosis in a patient with hematuria, especially in the setting of abdominal or flank pain, fatigue, anorexia, or weight loss.

## Figures and Tables

**Image:**
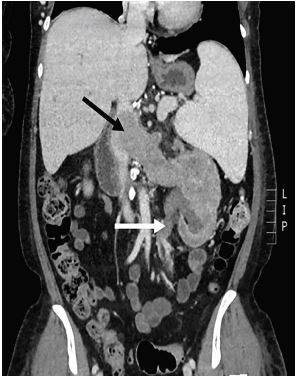
Coronal view of Computed Tomography of abdomen/pelvis with intravenous contrast demonstrating a necrotic mass in the left kidney with extension into the inferior vena cava (black arrow) and ureter (white arrow).

## References

[1] Cheville JC, Lohse CM, Zincke H (2003). Comparisons of outcome and prognostic features among histologic subtypes of renal cell carcinoma. Am J Surg Pathol.

[2] Luciani LG, Cestari R, Tallarigo C (2000). Incidental renal cell carcinoma—age and stage characterization and clinical implications: study of 1092 patients (1982–1997). Urology.

[3] Choyke PL, Amis ES, Bigongiari LR (2000). Renal cell carcinoma staging. ACR appropriateness criteria. Radiology.

[4] Marshall VF, Middleton RG, Holswade GR (1970). Surgery for renal cell carcinoma in the vena cava. J Urol.

[5] Lawrentschuk N, Gani J, Riordan R (2005). Multidetector computed tomography vs magnetic resonance imaging for defining the upper limit of tumour thrombus in renal cell carcinoma: a study and review. BJU Int.

